# Identification of novel pathways linking epithelial-to-mesenchymal transition with resistance to HER2-targeted therapy

**DOI:** 10.18632/oncotarget.7317

**Published:** 2016-02-11

**Authors:** Helen Creedon, Laura Gómez-Cuadrado, Žygimantė Tarnauskaitė, Jozef Balla, Marta Canel, Kenneth G. MacLeod, Bryan Serrels, Craig Fraser, Asier Unciti-Broceta, Natasha Tracey, Thierry Le Bihan, Teresa Klinowska, Andrew H. Sims, Adam Byron, Valerie G. Brunton

**Affiliations:** ^1^ Edinburgh Cancer Research Centre, Institute of Genetics and Molecular Medicine, University of Edinburgh, Edinburgh EH4 2XR, UK; ^2^ SynthSys, University of Edinburgh, Edinburgh EH9 3BF, UK; ^3^ AstraZeneca Oncology iMed, Alderley Park, Macclesfield SK10 4TG, UK

**Keywords:** resistance, breast cancer, EMT, HER2, proteomics

## Abstract

Resistance to human epidermal growth factor receptor 2 (HER2)-targeted therapies in the treatment of HER2-positive breast cancer is a major clinical problem. To identify pathways linked to resistance, we generated HER2-positive breast cancer cell lines which are resistant to either lapatinib or AZD8931, two pan-HER family kinase inhibitors. Resistance was HER2 independent and was associated with epithelial-to-mesenchymal transition (EMT), resulting in increased proliferation and migration of the resistant cells. Using a global proteomics approach, we identified a novel set of EMT-associated proteins linked to HER2-independent resistance. We demonstrate that a subset of these EMT-associated genes is predictive of prognosis within the ERBB2 subtype of human breast cancers. Furthermore, targeting the EMT-associated kinases Src and Axl potently inhibited proliferation of the resistant cells, and inhibitors to these kinases may provide additional options for the treatment of HER2-independent resistance in tumors.

## INTRODUCTION

The outlook for HER2-positive breast cancer patients has been revolutionized by the introduction of HER2-targeted agents, such as trastuzumab, pertuzumab and lapatinib [1]. However, both inherent and acquired resistance to these agents is a major clinical problem [2, 3]. Evidence from the Neo-ALTTO trial suggests that combined trastuzumab and lapatinib treatment is superior to use of either drug alone [4], and this, coupled with the almost universal development of acquired resistance, has driven efforts to develop novel therapies targeting this pathway. One approach has been the development of tyrosine kinase inhibitors that provide more effective inhibition of HER family signaling. One such drug is AZD8931, which is an equipotent, reversible inhibitor of signaling by EGFR, HER2 and HER3, having a unique and more potent profile of activity than lapatinib [5].

Numerous different mechanisms of acquired resistance to HER2-directed therapy have been identified. They frequently involve changes in HER2 expression or structure, but the development of HER2-independent strategies for activating survival pathways have also been widely reported [2, 3]. These pathways likely represent clinically relevant resistance mechanisms and suggest that novel methods of targeting the HER receptor family alone may not be sufficient to overcome resistance.

Using AZD8931- and lapatinib-resistant HER2-over-expressing breast cancer cell lines, we have identified that an epithelial-to-mesenchymal transition (EMT) is commonly associated with resistance. EMT is the name given to an evolutionarily conserved process in which epithelial cells lose cell-cell contacts and acquire a migratory mesenchymal phenotype accompanied by distinct changes in gene expression [6]. EMT has been linked to both chemo- and radio-resistance and resistance to targeted agents [7, 8]. In this study, we identified a common set of EMT-associated proteins that were linked to HER2-independent mechanisms of resistance to the HER2-directed drugs, and we provide evidence that targeting EMT-associated kinases Src and Axl may provide additional options for the treatment of resistant tumors.

## RESULTS

### Generation of AZD8931- and lapatinib-resistant breast cancer cell lines

We generated SKBR3 and BT474 cell lines that were resistant to AZD8931 or lapatinib by maintenance in increasing concentrations of either drug. Three AZD8931-resistant SKBR3 clones (SKBR3-AZDRa, SKBR3-AZDRb and SKBR3-AZDRc), two lapatinib-resistant SKBR3 clones (SKBR3-LAPRa and SKBR3-LAPRb) and two lapatinib-resistant BT474 clones (BT474-LAPRa and BT474-LAPRb) were selected. Despite increasing the maximum concentration of AZD8931 or lapatinib to 20 μM, the IC_50_ of the drugs in the respective resistant cell lines was not reached (Table 1). In addition, short-term treatment of the lapatinib-resistant cell lines with AZD8931 had no effect on their proliferation, and short-term treatment of the AZD8931-resistant cell lines with lapatinib had no effect on their proliferation, demonstrating cross-resistance between the two drugs (Table 1).

**Table 1:** Generation of AZD8931- and lapatinib-resistant SKBR3 and BT474 cell lines

Cell line	IC_50_ (μM)	
	AZD8931	Lapatinib
SKBR3	0.46	0.07
SKBR3-AZDRa	> 20	> 20
SKBR3-AZDRb	> 20	> 20
SKBR3-AZDRc	> 20	> 20
SKBR3-LAPRa	> 20	> 20
SKBR3-LAPRb	> 20	> 20
BT474	0.36	0.06
BT474-LAPRa	> 20	> 20
BT474-LAPRb	> 20	> 20

AlamarBlue cell viability assays were used to generate IC_50_ values for AZD8931 and lapatinib following treatment of cells with escalating drug concentrations for 72 hours.

**Figure 1:**
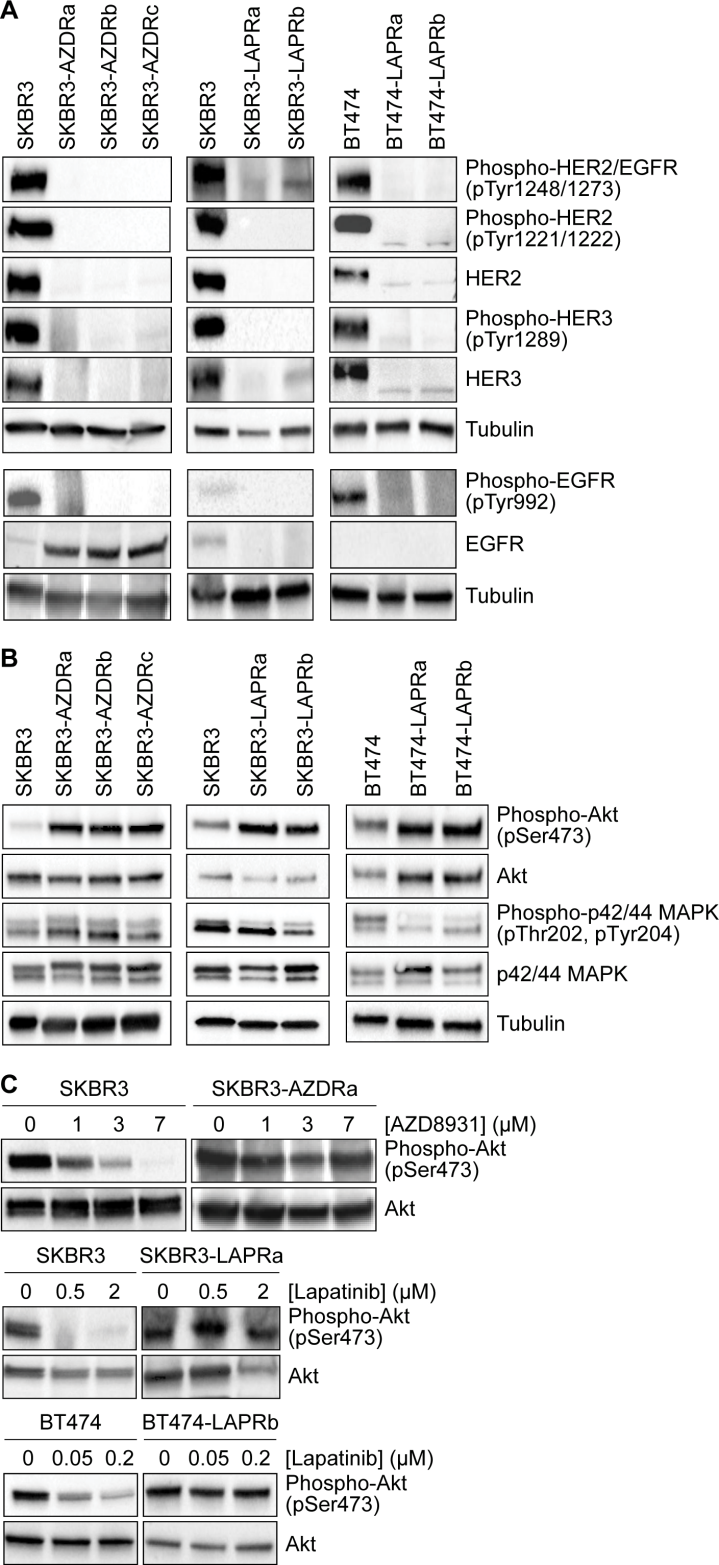
Loss of HER family signaling in AZD8931- and lapatinib-resistant cell lines (**A** and **B**) Whole cell lysates from untreated SKBR3 and BT474 parental and resistant cells were immunoblotted as indicated. Tubulin was used as a loading control. (**C**) Parental and resistant cells were treated for 12 hours with increasing concentrations of AZD8931 or lapatinib and then whole cell lysates were prepared and immunoblotted for phospho-Akt and Akt.

Resistance to both lapatinib and AZD8931 was associated with reduced expression and phosphorylation of HER2 and HER3 (Figure 1A). Both parental cell lines expressed very low levels of EGFR, and resistance to AZD8931 was associated with increased EGFR expression, which was not observed in the lapatinib-resistant cell lines. Furthermore, increased expression of EGFR in the AZD8931-resistant cell lines was not associated with increased phosphorylation on Tyr992, a recognized autophosphorylation site (Figure 1A).

Activation of the phosphatidylinositol 3-kinase (PI3K)/Akt and mitogen-activated protein kinase (MAPK) pathways are major downstream read-outs of HER family activity. Although HER family signaling was compromised in the resistant cell lines, there was a marked increase in Akt signaling in the AZD8931- and lapatinib-resistant cell lines, while more modest and varied effects were seen on MAPK signaling in the different cell lines (Figure 1B). Treatment with AZD8931 or lapatinib inhibited the activation of Akt in the parental cells, but sustained activation of the pathway was seen in the respective resistant cell lines, even at high concentrations of either AZD8931 or lapatinib (Figure 1C). Thus, the resistant cells have acquired the ability to activate the PI3K/Akt pathway in a HER family-independent manner.

### Multiple signaling pathways are altered in HER2-targeted drug-resistant cells

**Figure 2:**
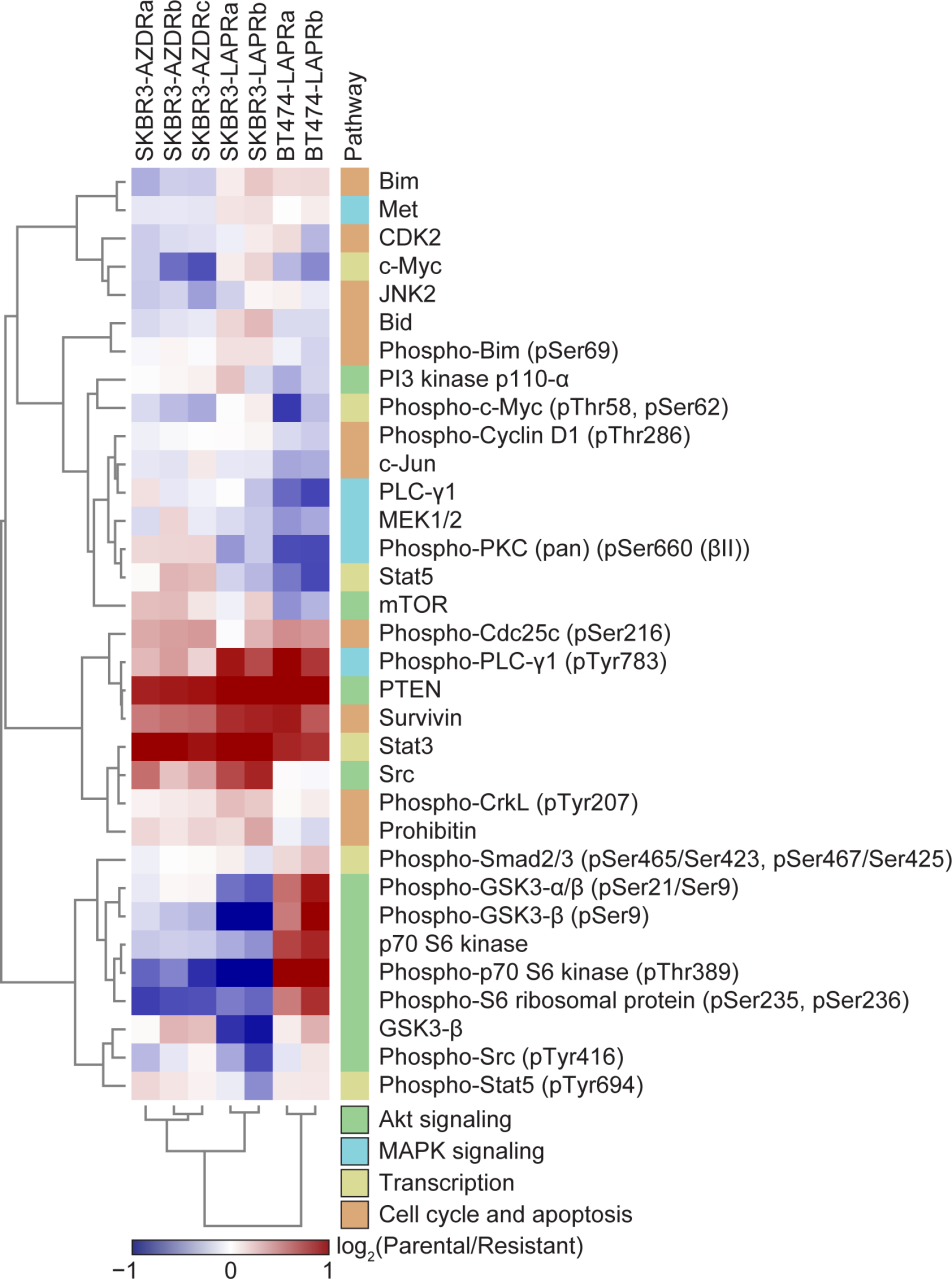
Signaling changes in AZD8931- and lapatinib-resistant cell lines Levels of signaling proteins and phosphoproteins in whole cell lysates from SKBR3 and BT474 parental and resistant cells were determined by RPPA. Normalized intensity values were scaled to respective parental intensity values and subjected to hierarchical clustering analysis. Heat map displays the protein or phosphoprotein enrichment in each resistant cell line relative to the respective parental cell line (red, up-regulated in parental cells; blue, up-regulated in resistant cells; log_2_ transformed). Relevant signaling pathways are annotated with a color bar (right).

To explore further the signaling changes associated with the development of resistance, we carried out reverse phase protein array (RPPA) analysis on the parental and resistant cells using a panel of antibodies covering a number of signaling pathways linked to cancer phenotypes (Supplementary Table S1). This revealed several changes in protein expression and phosphorylation in the resistant cell lines (Figure 2). Most notably, there was a striking reduction in expression of PTEN, Stat3 and survivin in all the resistant cell lines, with more modest and varied reductions in PLC-γ1 Tyr783 phosphorylation and Cdc25c Ser216 phosphorylation. There were also both cell line-specific and drug-specific changes in the resistant cells. For example, a reduction in phosphorylation of GSK3-β, p70 S6 kinase and S6 ribosomal protein was only seen in the lapatinib-resistant BT474 lines and not the lapatinib-resistant SKBR3 lines. In contrast, lapatinib resistance was associated with decreased expression of Bim and Met, while moderate increases in both these proteins were observed in the AZD8931-resistant cells. In addition, up-regulation of Stat5 and PKC Ser660 phosphorylation in the lapatinib-resistant cells was accompanied by slight down-regulation in the AZD8931-resistant cells. Such analysis provides insights into the complexities of signaling pathway deregulation in the resistant cells but also highlights common changes such as loss of PTEN, indicating that, as previously described, the PI3K/Akt pathway may be a common driver in resistance to HER2-targeted therapies.

### Resistance to AZD8931 and lapatinib is associated with an epithelial-to-mesenchymal transition

**Figure 3:**
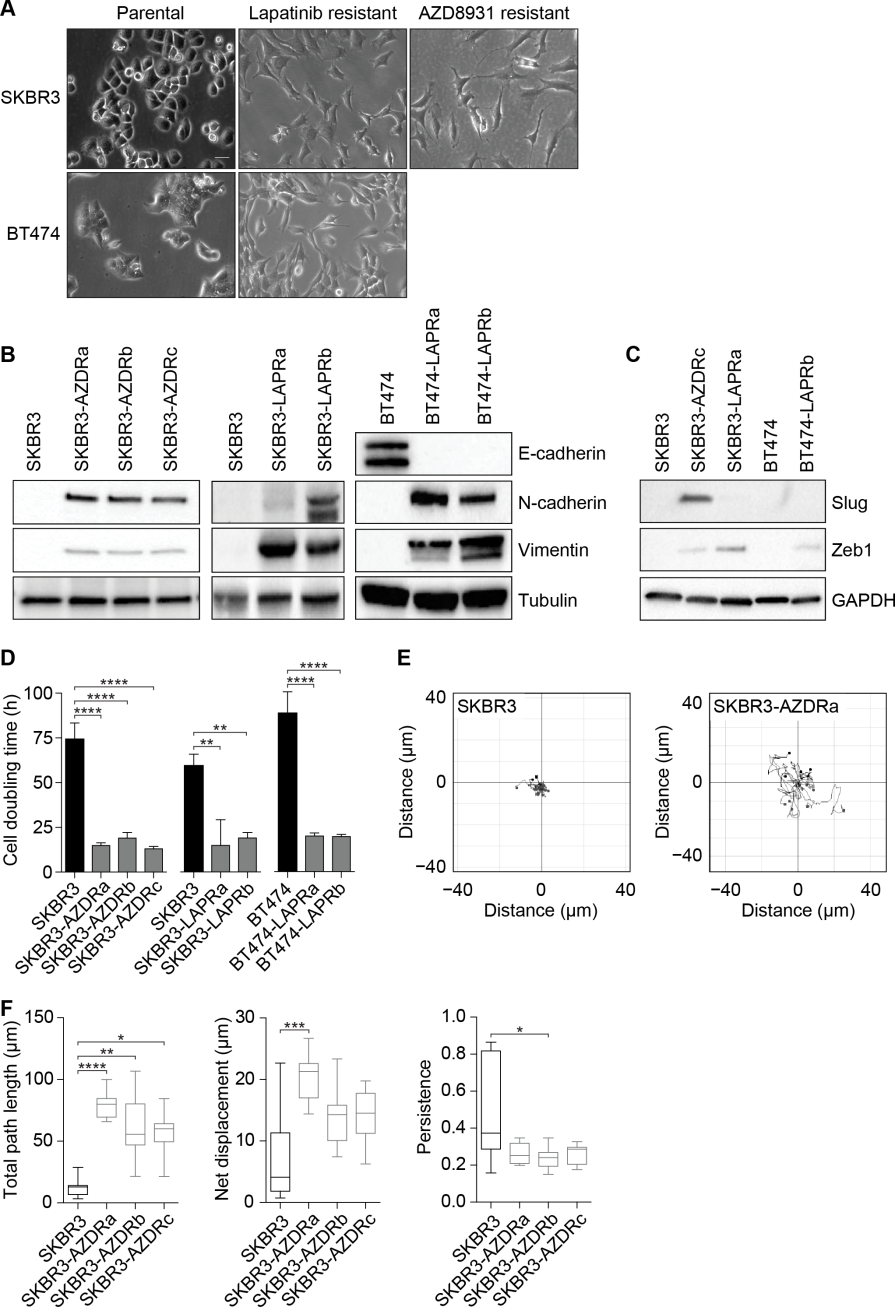
Resistance to AZD8931 is associated with an epithelial-to-mesenchymal transition (**A**) Phase-contrast images of parental and resistant SKBR3 and BT474 cell lines. Scale bar, 50 μm. (**B**) Whole cell lysates from parental and resistant SKBR3 and BT474 cell lines were immunoblotted for E-cadherin, N-cadherin and vimentin. Tubulin was used as a loading control. (**C**) Whole cell lysates from parental and resistant SKBR3 and BT474 cell lines were immunoblotted for Slug and Zeb1. GAPDH was used as a loading control. (**D**) Cell doubling times for the parental and resistant SKBR3 and BT474 cell lines. Results are mean ± s.d. (*n* = 3). (**E** and **F**) SKBR3 cells were seeded into six-well plates and, 24 hours later, images were recorded every 15 minutes for 16 hours. Representative images of individual tracks of parental (SKBR3; left) and AZD8931-resistant (SKBR3-AZDRa; right) cells are plotted (E). Total path length (accumulated distance; left), net displacement (Euclidean distance; middle) and directional persistence of migration (net displacement/total path length; right) were determined for parental and AZD8931-resistant SKBR3 cell lines (F). Box-and-whisker plots show the median (line), 25th and 75th percentiles (box) and 5th and 95th percentiles (whiskers) (*n* = 9) and are representative of three independent experiments. *p < 0.05, **p < 0.01, ***p < 0.001, ****p < 0.0001; one-way ANOVA with Tukey's *post hoc* correction (D), Kruskal–Wallis test with Dunn's *post hoc* correction versus parental cells (F).

One striking feature of both the AZD8931- and lapatinib-resistant cells was their distinctive mesenchymal appearance. The resistant cells had lost cell-cell contacts and acquired a spindle-like morphology (Figure 3A). In support of the resistant cells having undergone an EMT, we saw expression of the mesenchymal markers N-cadherin and vimentin in the AZD8931- and lapatinib-resistant SKBR3 cells but not the parental SKBR3 cells (Figure 3B). Loss of E-cadherin is also considered a hallmark of EMT, but SKBR3 cells contain a genetic deletion in *CDH1*, the E-cadherin-encoding gene, resulting in absent expression. In the BT474 cells, however, lapatinib resistance was associated with a loss of E-cadherin and concomitant expression of both N-cadherin and vimentin (Figure 3B). To understand potential drivers of the EMT phenotype, we analyzed the expression of key transcriptional regulators of EMT, Slug and Zeb1. Slug was over-expressed only in the AZD8931-resistant SKBR3 cells, whereas Zeb1 was expressed in all the AZD8931- and lapatinib-resistant BT474 and SKBR3 cells (Figure 3C). Thus, increased expression of Zeb1 provides a potential common mechanism whereby EMT is induced in the resistant cells.

The phenotypic change in the resistant cells was accompanied by a dramatic acceleration in the growth rate of the resistant cell lines compared to the parental line. The cell doubling time was significantly longer in the parental SKBR3 cell line compared to all three AZD8931-resistant cell lines. A similar reduction in doubling time was seen in the lapatinib-resistant SKBR3 and BT474 lines (Figure 3D).

Induction of EMT is associated with a more motile phenotype, and cell migration assays revealed that the distance travelled by individual cells was increased in all three AZD8931-resistant cell lines, with SKBR3-AZDRa being the most motile of the resistant cell lines (Figure 3E, 3F). Persistence of cell migration was reduced in the resistant cell lines, although this only reached statistical significance for SKBR3-AZDRb cells, suggesting a less directional mode of migration than the parental cells (Figure 3F).

### Global proteomic analysis identifies regulators of EMT in AZD8931-resistant cells

**Figure 4:**
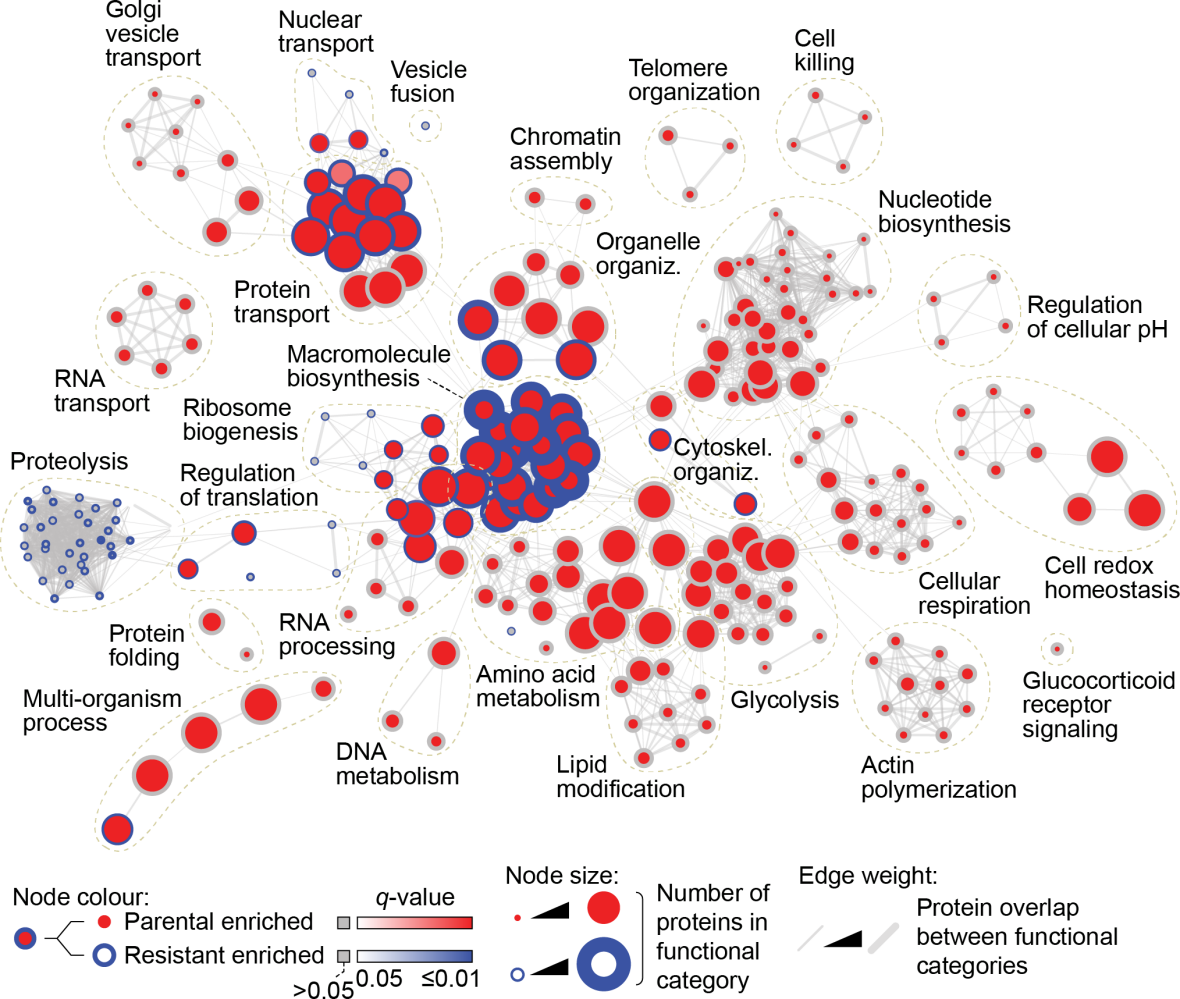
Proteomic analysis of parental and AZD8931-resistant SKBR3 cells Functional enrichment network analysis of proteins significantly differentially expressed between parental SKBR3 and AZD8931-resistant SKBR3-AZDRc cells (*p* < 0.05, one-way ANOVA), as determined by label-free quantitative MS. Nodes (circles) represent over-represented functional categories (GO biological processes; *p* < 0.05, hypergeometric test with Benjamini–Hochberg *post hoc* correction). Node border color intensity (blue) indicates significance of over-representation in resistant cells; node center color intensity (red) indicates significance of over-representation in parental cells. Nodes with blue borders and red centers represent functional categories over-represented in both parental and resistant cells. Node size indicates the number of differentially expressed proteins assigned to a given functional category. Edges (gray lines) connect GO biological processes with proteins in common; edge weight indicates degree of overlap between functional categories.

To identify possible regulators and markers of EMT that may be linked to resistance, we carried out label-free quantitative mass spectrometry (MS) analysis of parental and AZD8931-resistant SKBR3 cell lysates. We quantified 615 proteins (with at least two peptides) that were significantly differentially regulated between cell lines (*p* < 0.05) (Supplementary Table S2). Comparisons of the measured protein abundances showed a high positive correlation between all three biological replicates (*ρ* ≥ 0.99), indicating reproducible protein quantification by MS (Supplementary Figure S1). To interrogate the functional landscape of protein expression in parental and resistant cells, we analyzed the enrichment of cellular functions associated with the differentially regulated proteins (Supplementary Table S3). Over-represented gene ontology (GO) terms describing biological processes were mapped onto a functional network that connected and clustered biological processes associated with shared proteins (Figure 4). The network revealed several clusters of GO terms over-represented in the set of proteins up-regulated in resistant cells. A proteolysis-related cluster contained a number of resistant-enriched terms and included sequestersome 1 (SQSTM1) and the E3 ubiquitin ligase NEDD4 (Figure 4, Supplementary Table S3), proteins that are known to play roles in EMT [9, 10]. A large number of clusters contained parental-enriched terms, such as an actin polymerization cluster, which included LIM domain and actin binding 1 (LIMA1; EPLIN), whose down-regulation leads to EMT [11] (Figure 4, Supplementary Table S3).

**Figure 5:**
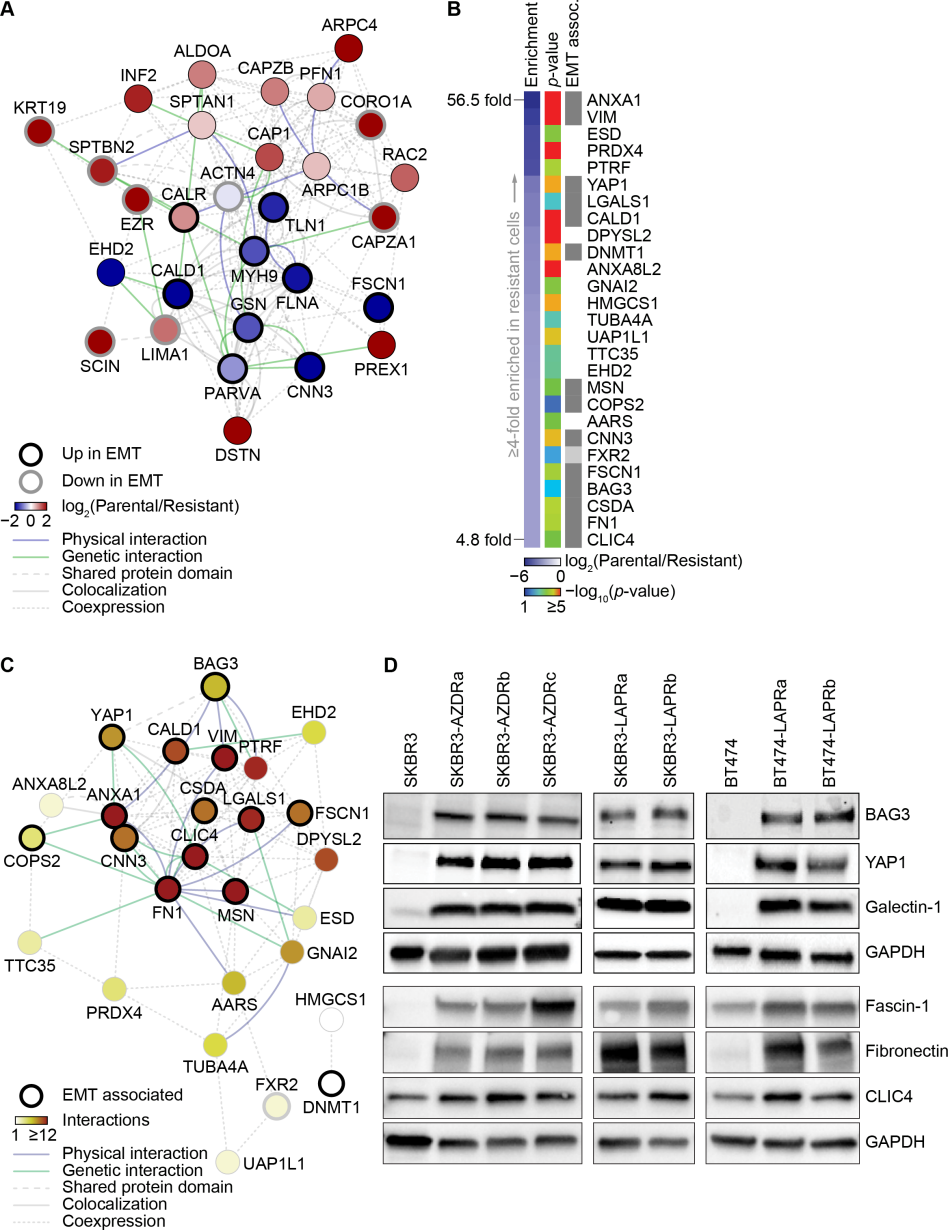
Changes in expression of EMT-associated proteins in AZD8931- and lapatinib-resistant cells (**A**) Interaction network analysis of proteins associated with over-represented actin polymerization-related functional categories (actin polymerization cluster, Figure 4). Nodes (circles) represent proteins; thick black node border indicates proteins reported to be up-regulated in EMT; thick gray node border indicates proteins reported to be down-regulated in EMT. Node color indicates log_2_-transformed protein fold enrichment (parental/resistant). Edges (lines) indicate various types of reported interactions. The network was clustered on the basis of the connectivity of the nodes. (**B**) Heat map generated from proteomic data from parental SKBR3 and AZD8931-resistant SKBR3-AZDRc cell lines, displaying proteins most up-regulated in resistant cells (by at least four fold; blue) alongside the significance of their enrichment (all *p* < 0.05, one-way ANOVA; rainbow). Gray boxes indicate proteins reported to be up-regulated during EMT; light gray box indicates a paralog associated with EMT. (**C**) Interaction network analysis of proteins enriched in AZD8931-resistant cells by at least four fold. Nodes represent proteins; thick black node borders indicate proteins associated with EMT; thick gray node border indicates a paralog associated with EMT. Edges indicate various types of reported interactions. Node color indicates number of interactions (degree) within the network; the network was clustered on the basis of the connectivity of the nodes. (**D**) Whole cell lysates from parental and resistant SKBR3 and BT474 cells were immunoblotted as indicated. GAPDH was used as a loading control.

Of the identified actin polymerization-associated proteins, 17 (59%) have been previously implicated in EMT (Figure 5A). Seven out of the eight proteins reported to be down-regulated in EMT were enriched in parental cells, whereas eight out of the nine proteins reported to be up-regulated in EMT were enriched in resistant cells, suggesting a link between EMT and AZD8931 resistance. Further analysis of the proteins most enriched in resistant cells showed that many of these (56% of proteins up-regulated by at least four-fold; *p* < 0.05) have previously been linked to EMT (Figure 5B). Interaction network analysis revealed that, of these resistant-enriched proteins, a highly interconnected core subnetwork was composed predominantly of EMT-associated proteins (Figure 5C). Importantly, western blotting confirmed up-regulation of a number of these proteins in both the AZD8931- and lapatinib-resistant SKBR3 and BT474 cell lines, including vimentin (VIM; Figure 3B), BAG3, YAP1, galectin-1 (LGALS1), fascin-1 (FSCN1), fibronectin (FN1) and CLIC4 (Figure 5D).

### Expression of EMT markers is associated with poor prognosis in HER2-positive tumors

**Figure 6:**
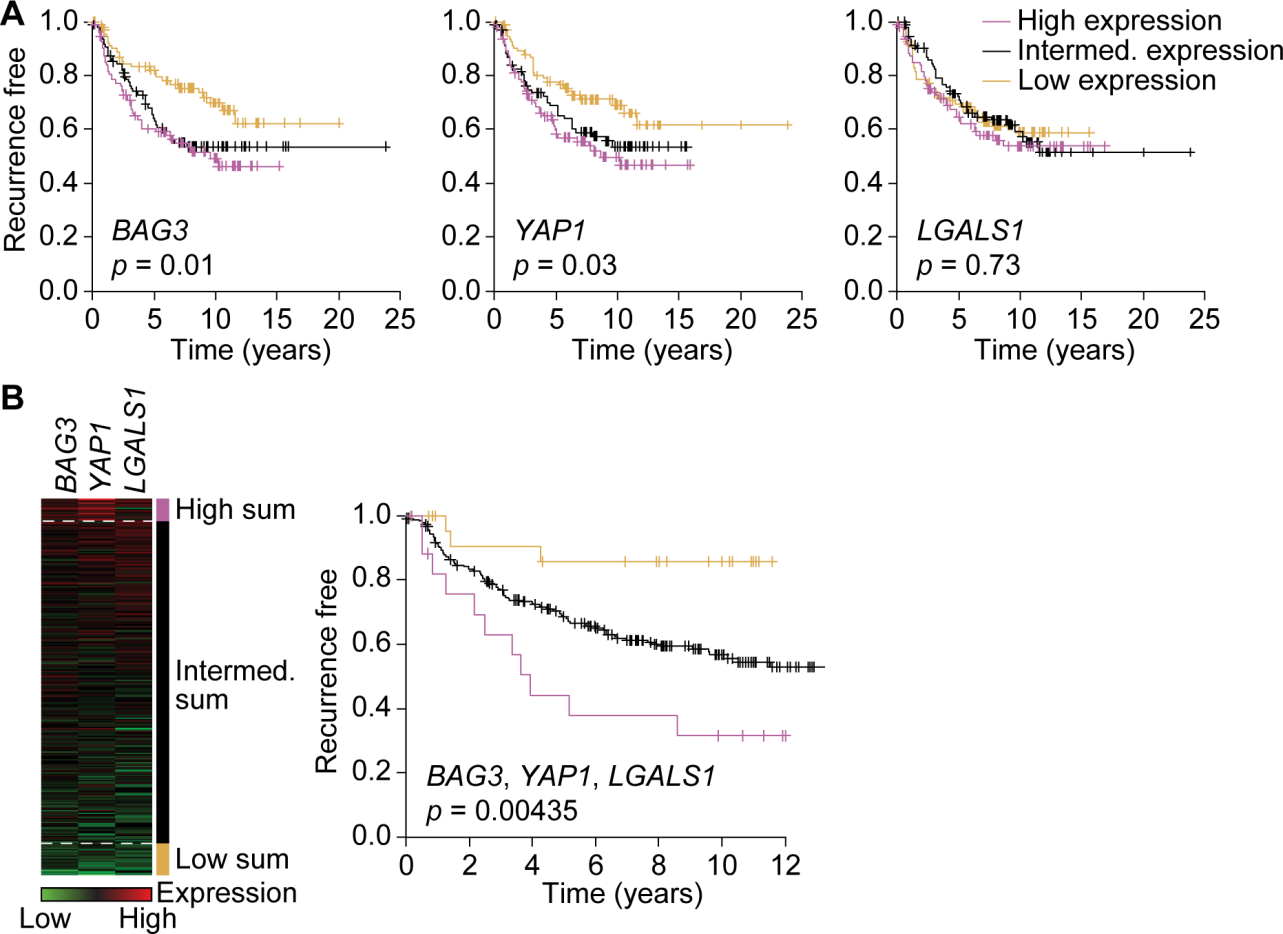
Expression of BAG3, YAP1 and galectin-1 is associated with poor prognosis (**A** and **B**) Kaplan–Meier analyses of 289 human breast cancers of the ERBB2 subtype. Time to recurrence is plotted in tertiles for high (purple, *n* = 96), intermediate (intermed.; black, *n* = 97) and low (gold, *n* = 96) expressors of *BAG3* (left), *YAP1* (middle) and *LGALS1* (right) (A). Patients with the highest (purple) and lowest (gold) sum of mean-centered gene expression of *BAG3*, *YAP1* and *LGALS1* have the worst and best prognosis, respectively (B). Significance was determined by log-rank test.

Although we were not able to assess whether the EMT-associated proteins over-expressed in the resistant cells represent markers of resistance for patients, we found that high gene expression of *BAG3* and *YAP1*, but not *LGALS1*, were significantly associated with poor prognosis in ERBB2-subtype tumors (Figure 6A). The expression of these genes was correlated across samples, and we found the sum of the three genes to be a predictor (Figure 6B). These samples were collected at diagnosis, so it seems that these EMT markers may identify a subset of patients whose tumors have *de novo* resistance to HER2-targeted therapy. Although *LGALS1* gene expression alone was not significantly associated with prognosis, immunohistochemical analysis of galectin-1 expression in human breast cancer has shown that it is expressed in both tumor cells and tumor-associated fibroblasts [12]. Further analysis of tumor cell-associated galectin-1 expression in ERBB2 tumors is therefore required to determine whether this is associated with poor prognosis.

### Targeting EMT-associated drug resistance

Although our proteomic analysis identified potential markers of resistance, it did not identify actionable kinases that could provide potential treatment options for the resistant cells. However, we have previously reported increased expression and/or activity of a number of EMT-associated tyrosine kinases, including Src and Axl, in both lapatinib- and AZD8931-resistant cells [2]. As both Src and Axl inhibitors are under clinical development, we asked whether these kinases were important for the EMT-associated drug resistance. Treatment with the Src family kinase inhibitor dasatinib potently inhibited the proliferation of the AZD8931-resistant but not the parental SKBR3 cells (Table 2). We also used eCF506, which is a highly selective Src family inhibitor with selectivity over other kinases such as c-Abl, PDGFRα and c-Kit (Footnote 1). eCF506 inhibited the growth of the resistant but not the parental SKBR3 cells (Table 2). Treatment with the Axl inhibitor foretinib also potently inhibited the proliferation of the resistant but not the parental SKBR3 cells (Table 3). Thus, both Src and Axl kinase activities are important for the EMT-associated drug resistance, and inhibitors to these kinases may provide alternative treatment options in tumors that are resistant to HER2-targeted therapies that have undergone EMT.

**Table 2:** Increased sensitivity of resistant cells to Src inhibitors

Cell line	IC_50_ (μM)	
	Dasatinib	eCF506
SKBR3	> 20	> 20
SKBR3-AZDRa	0.042	0.212
SKBR3-AZDRb	0.062	0.799
SKBR3-AZDRc	0.029	0.639

PrestoBlue cell viability assays were used to generate IC_50_ values for dasatinib and eCF506 following treatment of cells with escalating drug concentrations for 72 hours.

**Table 3:** Increased sensitivity of resistant cells to Axl inhibitors

Cell line	IC_50_ (μM)
	Foretinib
SKBR3	> 20
SKBR3-AZDRc	2.01
BT474	> 20
BT474-LAPRb	0.43

AlamarBlue cell viability assays were used to generate IC_50_ values for foretinib following treatment of cells with escalating drug concentrations for 72 hours.

## DISCUSSION

Resistance to HER2-targeted therapies is a major clinical problem, and understanding the mechanism driving resistance is required to provide new treatment options. In our models of acquired resistance to both lapatinib and AZD8931, we found reduced HER2 signaling accompanied by activation of Akt. However, other studies have shown persistent HER2 signaling following the development of HER2-targeted therapy resistance, with activation of PI3K signaling, loss of PTEN, increased IGF1R expression and enhanced Src activity all having been linked to resistance. These same pathways are active in our resistant cell lines [2], suggesting that certain signaling pathways are activated following the development of resistance, irrespective of the primary effects on HER2 signaling. To date, clinical approaches to overcome resistance have predominantly focused on utilizing different strategies to target HER2 signaling, yet this overlap of HER2-dependent and HER2-independent resistance mechanisms may explain why, although frequently initially effective, novel methods of targeting HER2 have had limited longer-term efficacy, with the development of resistance still remaining inevitable.

We also identified several potential novel markers of AZD8931 and lapatinib resistance, including reduced phosphorylation of PLCɣ. The SH2 domain of PLCɣ is phosphorylated on Tyr783 by EGFR [13], and therefore phosphorylation of PLCɣ at this site can be used as a biological readout of EGFR activity. This is consistent with the reduction in EGFR autophosphorylation in the resistant cells identified by western blotting. We also observed a significant reduction in STAT3 expression in our AZD8931- and lapatinib-resistant cell lines, although previous reports have suggested that an active STAT3 feedback loop is important for driving drug resistance [14]. The activation status of STAT3 was not determined in our analysis.

Cells which have undergone EMT are widely reported to be resistant to conventional anti-cancer therapies, including chemotherapy, radiotherapy and targeted therapies including HER2-targeted therapies [15]. In preclinical *in vitro* models, genetic modulation of transcriptional EMT drivers and the reversion to an epithelial phenotype can restore sensitivity to HER2-targeted agents [16–18]. Here, we show that expression of a transcriptional regulator of EMT, Zeb1, is substantially increased in all the resistant cell lines, consistent with a Zeb1-mediated induction of EMT in the resistant cells. Induction of EMT is also linked to the acquisition of stem cell properties [19], and resistance to trastuzumab has been associated with expansion of a cancer stem cell population with EMT properties [18, 20]. Moreover, induction of EMT in HER2-driven tumors via expression of an activating PI3K mutation has been associated with increased expression of cancer stem cell markers and resistance to trastuzumab and lapatinib [21].

We have identified that over-expression of *BAG3*, *YAP1* and *LGALS1* was associated with poor prognosis in HER2-positive breast cancers. However, further work is required to identify whether such EMT gene signatures in clinical samples associate with resistance to HER2-targeted therapies. Induction of EMT in both *in vitro* and *in vivo* models is also associated with *de novo* resistance to HER2-targeted therapies [18, 21], and it is possible that these EMT gene signatures may also provide potential prognostic biomarkers to aid the stratification of patient treatment.

Targeting EMT-associated resistance may also be a useful therapeutic approach. For example, small molecule inhibitors of Axl have activity in lapatinib-resistant breast cancer models [22], and our data show that both AZD8931- and lapatinib-resistant cells are more sensitive than the parental cells to foretinib, which inhibits Axl tyrosine kinase activity. We also show that the resistant cells are more sensitive to Src kinase inhibitors, which suggests that Axl and Src inhibitors, both of which are in clinical development, may have utility in the treatment of resistant tumors in which the Axl and Src signaling pathways are up-regulated. Importantly, this provides further treatment options for tumors that have developed HER2-independent mechanisms of resistance that would not benefit from further HER2-directed therapies, as is current clinical practice. As it is rarely mandatory to re-biopsy tumors at the time of entry into clinical trials, patients who have developed resistance and whose tumors no longer express HER2 risk being exposed to the toxicity of treatments that might not be anticipated to be effective. Moving forward, it will be important to identify the most clinically relevant markers linked with EMT-associated resistance and determine their expression upon relapse to HER2-targeted therapies to guide future treatment.

## MATERIALS AND METHODS

### Cell culture

Human breast cancer cell lines SKBR3 and BT474 were purchased from the American Type Culture Collection. SKBR3 cells were grown in Dulbecco's modified Eagle's medium (DMEM) supplemented with 2 mM L-glutamine, 1% penicillin-streptomycin and 10% FCS (all Thermo Fisher Scientific). BT474 cells were grown in RPMI-1640 supplemented with 2 mM L-glutamine, 1% penicillin-streptomycin and 10% FCS. Cells were maintained at 37°C in a humidified atmosphere containing 5% CO_2_. A Leica DM IL LED microscope in conjunction with a QImaging Retiga EXi Fast 1394 camera was used to capture phase-contrast images of cells.

### Generation of resistant cell lines

Lapatinib-resistant cells were established by culturing cells in complete medium supplemented with escalating concentrations of lapatinib (0.04–5 μM; SelleckChem). Cells were then maintained in 5 μM lapatinib. AZD8931-resistant cells were established by culturing cells in complete medium supplemented with escalating concentrations of AZD8931 (0.0067–0.67 μM; provided by AstraZeneca) and maintained in 0.67 μM AZD8931. Prior to drug treatment studies, the cells were grown for one week in the absence of drug.

### Cell viability assays

Cells were seeded in 96-well plates and allowed to attach for 24 hours. Escalating doses of lapatinib, AZD8931, dasatinib (Synkinase), foretinib (SelleckChem) or eCF506 (Footnote 1) prepared in DMSO were then added. After 72 hours, alamarBlue or PrestoBlue (Thermo Fisher Scientific) cell viability reagent was added and fluorescence measured after a further 60 minutes. Mean values were calculated from six replicate wells and normalized against the mean value of the vehicle (DMSO)-treated wells, and IC_50_ values were generated using Prism (GraphPad).

### Western blotting

Cells were washed with PBS and then lysed in RIPA buffer supplemented with cOmplete ULTRA protease inhibitor and PhosSTOP phosphatase inhibitor cocktails (Roche). Cleared lysates were resolved by SDS-PAGE. Primary antibodies used for western blotting were as follows: phospho-HER2/EGFR (phospho-tyrosine (pTyr)-1248/1273), phospho-HER2 (pTyr1221/1222), HER2, phospho-HER3 (pTyr1289), HER3, phospho-EGFR (pTyr992), EGFR, phospho-Akt (phospho-serine (pSer)-473), Akt, phospho-p44/42 MAPK (phospho-threonine (pThr)-202, pTyr204), p44/42 MAPK, E-cadherin, Slug, vimentin, YAP1 (all 1:1000; Cell Signaling Technologies), BAG3, CLIC4, fibronectin (all 1:1000; Abcam), Zeb1 (1:2000; Abcam), galectin-1, N-cadherin (all 1:1000; BD Biosciences), fascin-1 (1:1000; Santa Cruz Biotechnology), α-tubulin (1:3000; Sigma-Aldrich), GAPDH (1:2500; Thermo Fisher Scientific).

### RPPA analysis

Cells, in biological triplicate, were washed with PBS and lysed in 1% Triton X-100, 50 mM HEPES (pH 7.4), 150 mM sodium chloride, 1.5 mM magnesium chloride, 1 mM EGTA, 100 mM sodium fluoride, 10 mM sodium pyrophosphate, 1 mM sodium vanadate, 10% glycerol, supplemented with cOmplete ULTRA protease inhibitor and PhosSTOP phosphatase inhibitor cocktails. Cleared lysates were serially diluted to produce a dilution series comprising four serial two-fold dilutions of each sample, which were spotted onto nitrocellulose-coated slides (Grace Bio-Labs) in technical triplicate under conditions of constant 70% humidity using the Aushon 2470 array platform (Aushon Biosystems). Slides were hydrated in blocking buffer (Thermo Fisher Scientific) and then incubated with validated primary antibodies (all 1:250; Supplementary Table S1). Bound antibodies were detected by incubation with anti-rabbit DyLight 800-conjugated secondary antibody (New England BioLabs). An InnoScan 710-IR scanner (Innopsys) was used to read the slides, and images were acquired at the highest gain without saturation of the fluorescence signal. The relative fluorescence intensity of each sample spot was quantified using Mapix software (Innopsys).

The linear fit of the dilution series of each sample was determined for each primary antibody, from which median relative fluorescence intensities were calculated. Signal intensities were normalized by global sample median normalization [23]. Only primary antibodies with normalized signal intensities at least 1.5 times the value of the secondary antibody alone in at least one sample were included in the analysis to exclude data derived from weak or non-specific signals.

### MS analysis

Parental SKBR3 and AZD8931-resistant SKBR3-AZDRc cell pellets (1 mg protein equivalent), in biological triplicate, were reconstituted in 8 M urea, 25 mM ammonium bicarbonate, 20 mM dithiothreitol to denature and reduce the samples (30 minutes), followed by alkylation with 50 mM iodoacetamide (1 hour). Samples were digested with 10 μg trypsin overnight at room temperature. Peptide extracts were then cleaned on an SPE reverse-phase Bond Elut LMS cartridge (Agilent) and evaporated to dryness. Peptides were re-suspended in 2.5% acetonitrile, 0.1% formic acid in water to give a final concentration of 1 μg/μL.

Peptides (4 μg) were subjected to nano-scale high-performance liquid chromatography (HPLC)-MS using a nano-pump (Dionex Ultimate 3000; Thermo Fisher Scientific) with a 300 μm × 5 mm pre-column (5 μm particle size, Acclaim PepMap; Thermo Fisher Scientific) connected to a 75 μm × 50 cm column (3 μm particle size, Acclaim PepMap; Thermo Fisher Scientific). HPLC was coupled on-line to a Q Exactive instrument (Thermo Fisher Scientific) controlled by Xcalibur (Thermo Fisher Scientific; version 3.0.63) using Tune (version 2.3, build 1765). Samples were analyzed on a two-hour gradient using data-dependent analysis with one 70 k-resolution survey scan followed by the top five MS/MS scans at 17.5 k resolution.

Peak lists were generated using MSConvert (ProteoWizard; version 3.0.4462). MS data were searched against the National Center for Biotechnology Information (NCBI) protein database (*Homo sapiens*; 34,284 sequences; downloaded 12 January 2011) using Mascot software (version 2.4; Matrix Science). Up to two missed tryptic cleavage sites per peptide were permitted. Variable methionine oxidation and fixed cysteine carbamidomethylation modifications were allowed. Precursor and product ion mass tolerances were set to 10 ppm and 0.05 amu, respectively. A final peptide score of at least 20 and *p* < 0.05 (MudPIT scoring) were required, which resulted in a global false discovery rate of less than 1%.

Label-free quantification was performed using Progenesis LC-MS (version 4.1; Nonlinear Dynamics). Only MS peaks with a charge of 2+, 3+ or 4+ and the five most intense spectra within each feature were extracted from each LC-MS run for analysis. Normalization was first performed based on the sum of the ion intensities of these sets of multi-charged ions (2+, 3+, 4+). The associated unique peptide ion intensities for a specific protein were then summed to generate an abundance value, which was transformed using an ArcSinH function. The within-group means were calculated to determine the fold change between conditions, and the transformed data were used to calculate the *p*-values using one-way analysis of variance (ANOVA).

MS data were deposited in ProteomeXchange (http://www.proteomexchange.org) via the PRIDE partner repository (http://www.ebi.ac.uk/pride) with the dataset identifier PXD002057 (DOI: 10.6019/PXD002057).

### Hierarchical clustering analysis

Unsupervised hierarchical clustering analysis of normalized protein expression was performed on the basis of Pearson correlation using Cluster 3.0 (C Clustering Library, version 1.37) [24], computing distances using a complete-linkage matrix. Clustering results were visualized using Java TreeView (version 1.1.1) [25] and MultiExperiment Viewer (version 4.1.01) [26].

### Network analyses

Functional enrichment analysis was performed using BiNGO, assessing over-representation of GO terms describing biological processes using a hypergeometric test with Benjamini–Hochberg *post hoc* correction. Network clusters were generated from over-represented GO terms using Enrichment Map in Cytoscape (version 3.0.2) [27], with a Jaccard coefficient cutoff of 0.25, and manually annotated. Interaction networks were constructed using GeneMania, clustered using the yFiles Organic algorithm in Cytoscape, and network topology was analyzed from undirected graphs using NetworkAnalyzer [28].

### Cell migration assay

Cells were plated in duplicate at 3 × 10^3^ cells per well in a 12-well plate in DMEM containing 10% FCS and 10 mM HEPES. Random migration was monitored by time-lapse video microscopy over 16 hours on an Olympus scan^R screening station. Total path length (accumulated distance) and net displacement (Euclidean distance) of individual cells were calculated using ImageJ software (National Institutes of Health). Directional persistence of cell migration was calculated by dividing net displacement by total path length.

### Survival analysis of gene expression data

Gene expression levels of the EMT-associated proteins that were over-expressed in the resistant cells were assessed in 289 ERBB2-subtype primary breast tumors from a compendium of 2999 tumors integrated from 17 studies, as previously described [29]. Briefly, raw Affymetrix U133A/plus 2 .cel files were downloaded from the NCBI Gene Expression Omnibus (GSE12276, GSE21653, GSE3744, GSE5460, GSE2109, GSE1561, GSE17907, GSE2990, GSE7390, GSE11121, GSE16716, GSE2034, GSE1456, GSE6532, GSE3494, GSE19615) and cancer Biomedical Informatics Grid (geral-00143) repositories, summarized with Ensembl alternative CDF [30], normalized with RMA [31] and integrated using ComBat [32] to remove dataset-specific bias, as previously described [33]. The intrinsic molecular subtypes were assigned based upon the highest correlation to the intrinsic subtype centroids [34]. Survival analysis was performed using the *survival* R package [35].

## SUPPLEMENTARY FIGURES AND TABLES








