# Supplementary material for: Identification of novel pathways linking epithelial-to-mesenchymal transition with resistance to HER2-targeted therapy
Source: Oncotarget. 2016 Feb 11;7(10):11539–52. doi: 10.18632/oncotarget.7317 (PMC4905492; doi:10.18632/oncotarget.7317)
Supplement: Supplementary file 1 [file oncotarget-07-11539-s001.pdf]

# Identification of novel pathways linking epithelial-to-mesenchymal transition with resistance to HER2-targeted therapy

## Supplementary Materials

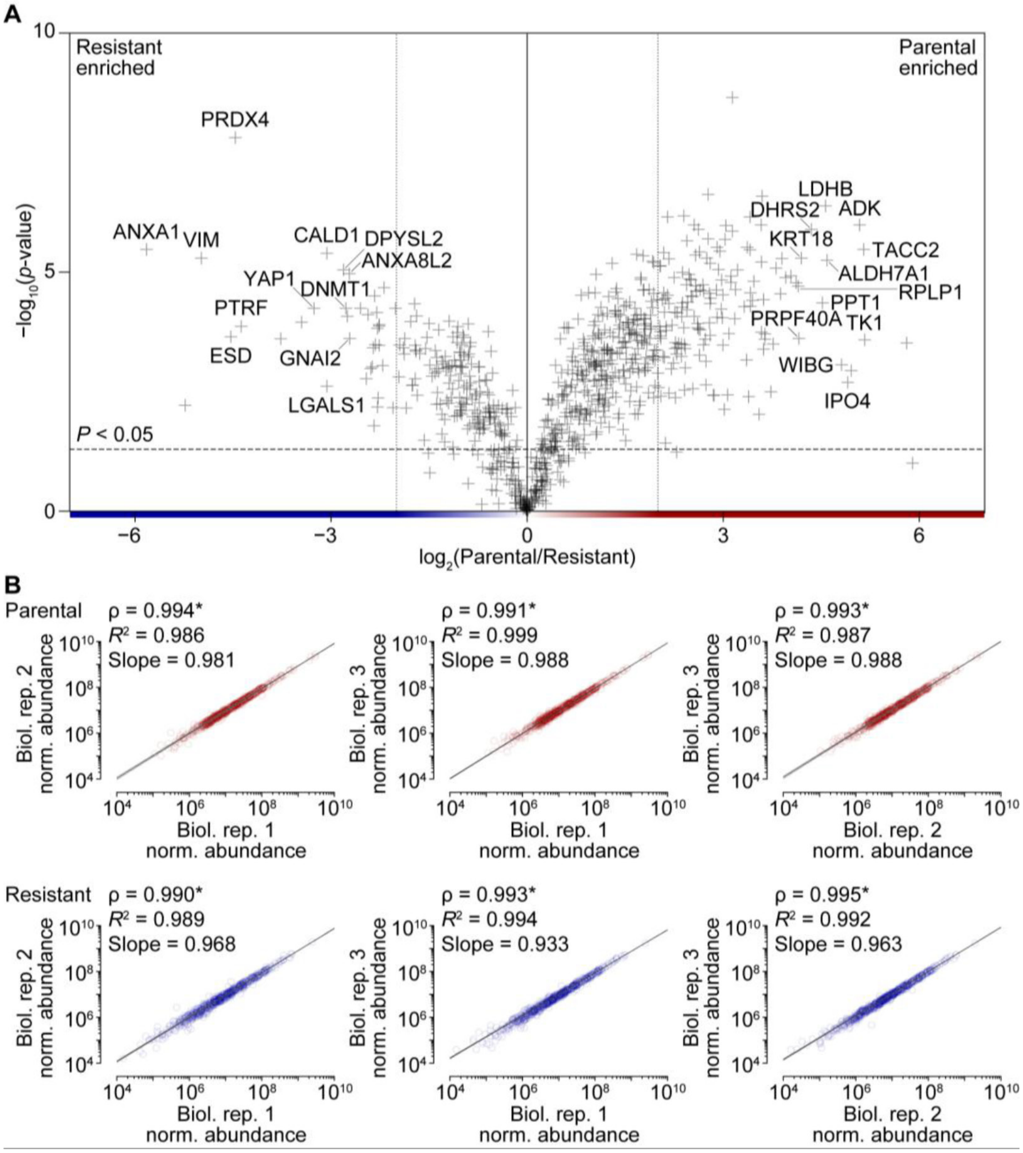


**Supplementary Figure S1: Reproducibility and distribution of MS data.** (**A**) Volcano plot of the distribution of all proteins confidently identified by MS in parental SKBR3 or AZD8931-resistant SKBR3-AZDRc cell lysates (795 proteins). Dashed horizontal line indicates threshold for significantly differentially expressed proteins ($p < 0.05$, one-way ANOVA); dotted vertical lines indicate up-regulation in parental (right) or resistant (left) cells by four fold. Color bar on *x*-axis indicates $\log_2$-transformed relative protein enrichment (parental/resistant). The 12 most enriched proteins are indicated with gene names for clarity (excluding proteins quantified with fewer than two peptides). All identified proteins are detailed in Supplementary Table S2. (**B**) Label-free MS quantification of parental or resistant cells was compared between biological triplicate analyses. Proteins quantified with at least two peptides that were significantly differentially expressed between cell lines (615 proteins) are displayed. Spearman correlation coefficients (ρ) are shown; asterisks indicate significant correlation ($p < 0.0001$). Log–log lines (black lines) and associated 95% confidence bands (gray lines, mostly obscured by black lines owing to tight fits) were calculated by nonlinear regression analysis (least squares fit). Goodness of fit ($R^2$) and slope best fit values are displayed for each pairwise comparison. Hougaard's measure of skewness was < 0.04 (almost linear) for all slope best fits.

**Supplementary Table S1: Antibodies used for RPPA.**

**Supplementary Table S2: Proteomic analysis of parental and AZD8931-resistant cells.** (**A**) Proteins identified by proteomic analysis of lysates of parental SKBR3 and AZD8931-resistant SKBR3-AZDRc cells. Proteins with fewer than two peptides used for quantification or with $p \geq 0.05$ (one-way ANOVA) were excluded from further differential analysis; these entries are shaded in gray. (**B**) Proteins up-regulated in parental cells as quantified by proteomic analysis of lysates of parental SKBR3 and AZD8931-resistant SKBR3-AZDRc cells ($p < 0.05$, one-way ANOVA). Shading indicates fold change ratio ≥ 4 (red). (**C**) Proteins up-regulated in resistant cells as quantified by proteomic analysis of lysates of parental SKBR3 and AZD8931-resistant SKBR3-AZDRc cells ($p < 0.05$, one-way ANOVA). Shading indicates fold change ratio ≤ 0.25 (blue).

**Supplementary Table S3: Functional enrichment analysis of proteins differentially regulated in parental and AZD8931-resistant cells.** (**A**) Functional categories (GO biological processes) enriched in proteins up-regulated in parental SKBR3 cells. (**B**) Functional categories (GO biological processes) enriched in proteins up-regulated in AZD8931-resistant SKBR3-AZDRc cells.
